# Patient-reported results of simultaneous direct anterior approach and posterolateral approach total hip arthroplasties performed in the same patients

**DOI:** 10.1186/s10195-021-00611-w

**Published:** 2021-11-13

**Authors:** Zhi Yang, Shuo Feng, Kai-Jin Guo, Guo-Chun Zha

**Affiliations:** Department of Orthopedic Surgery, The Affiliated Hospital of Xuzhou Medical University, No. 99 Huaihai West Road, Xuzhou, 221002 Jiangsu People’s Republic of China

**Keywords:** Direct anterior approach, Posterolateral approach, Patient-reported outcome measures, Total hip arthroplasty

## Abstract

**Background:**

Several studies have compared clinical results of the direct anterior approach (DAA) and the posterolateral approach (PLA) in total hip arthroplasty (THA); however, the effect of the surgical approach on outcome of THA remains controversial. Most of these studies used two distinct groups of patients, and THAs were performed by different surgeons, using different designs of prosthesis. These confounding factors may limit the strength of the conclusions. The purpose of this prospective, simultaneous bilateral randomized study was to investigate whether patients would perceive the difference between the direct anterior approach (DAA) and the posterolateral approach (PLA) after THA.

**Materials and methods:**

Among 20 patients scheduled to undergo same-day bilateral THA between October 2017 and August 2019, one hip was randomly assigned to DAA and the other to PLA. Patient-reported outcome measures [Hip disability and Osteoarthritis Outcome Score (HOOS), patients’ hip pain on mobilization] and physician-assessed measures [Harris Hip Score (HHS), operative time, intraoperative blood loss, cup abduction, cup anteversion, stem orientation, and incidence of complications (intraoperative fracture, nerve damage, incisional problem, or postoperative dislocation)] were compared.

**Results:**

All patients were followed up for 12 months. Hip pain was significantly less with DAA-THA compared with PLA-THA at postoperative 1, 3, and 7 days (*p* < 0.05). There was no clinical difference between DAA-THA and PLA-THA in terms of the VAS, HOOS, or HSS at 6 weeks and 3, 6, and 12 months postoperatively (*p* > 0.05). DAA-THA had a longer operative time and shorter length of incision compared with PLA-THA. There was no statistical difference between DAA-THA and PLA-THA in terms of intraoperative blood loss, cup abduction, cup anteversion, stem orientation, and perioperative complications (*p* > 0.05).

**Conclusions:**

This study demonstrates that DAA-THA and PLA-THA could provide comparable HHS and HOOS at all follow-ups. Compared with PLA-THA, DAA-THA is associated with less hip pain within postoperative 7 days and shorter incision length, but longer operative time.

**Level of evidence:**

Level I, therapeutic study.

*Trial registration* Chinese Clinical Trail Registry, ChiCTR1800019816. Registered 30 November 2018—retrospectively registered, http://www.chictr.org.cn/showproj.aspx?proj=30863

## Introduction

Total hip arthroplasty (THA) is a frequently used orthopedic surgery worldwide and has a high success rate in the treatment of hip disease [1]. Various approaches, including the posterolateral approach, lateral approach, direct anterior approach, and anterolateral approach, have been used for THA; however, the effect of the surgical approach on the outcome of THA remains controversial [2, 3].

Of these, the posterolateral approach (PLA) is the one most commonly used by surgeons [3], while the direct anterior approach (DAA) THA is now widely promoted as a true interneural and intermuscular approach that reduces muscle damage, decreases postoperative dislocation, and accelerates postoperative recovery, following advocacy by some surgeons and manufacturers [4, 5]. However, several studies have shown that early functional outcomes, especially objective locomotor parameters, are similar for both DAA-THA and PLA-THA [6], and even DAA is associated with a higher risk of complications, including femoral fractures [7], lateral femoral cutaneous nerve injury [8], and early revision [7, 9].

Although several studies have compared the clinical results of DAA and PLA, most of these studies used two distinct groups of patients and THAs were performed by different surgeons, using different designs of prosthesis [6, 10–12]. Comparison of DAA-THA’s true benefit should be evaluated with the same patient and surgeon, and identical design of the prostheses. In addition, these studies focused on the objective results assessed by surgeons (e.g., prosthesis position, muscle damage, blood loss, dislocation rate, etc.) or on surgeon-based outcome tools (e.g., Harris Hip Score) [13–16]. The outcome and implication of a surgical approach for THA should been evaluated from the patient’s perspective, so the patient-reported outcome measures (PROMs) are increasingly accepted as an important part of assessing outcomes after THA [17–19].

With this background, we conducted this prospective study to compare the PROMs after DAA and PLA in patients who underwent same-day simultaneous bilateral THAs with identical prostheses by the same surgeon, with DAA on one side and PLA on the other side.

## Materials and methods

From October 2017 to August 2019, we prospectively enrolled 20 patients (40 hips) with bilateral symmetrical end-stage femoral head osteonecrosis, who underwent bilateral simultaneous THAs using a uncemented cup and stem (Trilogy Acetabular Shell, CLS Spotorno stem; Zimmer, Warsaw, Indiana). In this study, patients were excluded if they had prior hip surgery, foot or ankle or knee disorders, dementia, or history of stroke, or they were older than 75 years of age or were classified as greater than grade II according to the American Society of Anesthesiologists (ASA). This study was approved by the Ethics Committee of Affiliated Hospital of Xuzhou Medical University (no. XYEY2014-xjs010-02). All methods were performed in accordance with the relevant guidelines and regulations, and all patients gave informed consent.

Randomization of DAA-THA or PLA-THA was accomplished using study numbers in sealed opaque envelope that was opened in the operating room before the skin incision was made. A computer program equally assigned all patients to receive one approach in one hip and the other in the contralateral hip. When DAA-THA was assigned as the first operation, PLA-THA was performed first in the next patient. This was constant for all patients. This study was performed according to the intention-to-treat principles. All patients successfully completed bilateral surgery.

All surgical procedures were performed by a single surgeon (G.C.Z.), using identical cementless prostheses under general anesthesia. DAA-THA and PLA-THA were performed in lateral decubitus position with the smallest incision possible. The surgeon (G.C.Z.) was specially trained and experienced in both approaches. Prior to the study, he had completed 120 cases of DDA–THA and 1000 cases of PLA-THA. The surgical techniques utilized the widely accepted standard DAA and PLA, and are consistent with the techniques described elsewhere [20–22]. Neither DAA nor PLA used intraoperative X-ray. No drainage was placed in either approach.

Patients started walking on the first postoperative day, and they progressed to full weight-bearing with a walker or crutches as tolerated; they were advised to use a walking aid for 4 weeks to prevent falls, and to avoid any dislocation-prone actions within 4 weeks after surgery, such as hyperextension and external rotation in DAA-THA, and hyperflexion and internal rotation in PLA-THA.

Patients were evaluated at the following timepoints: preoperatively, intraoperatively, and 1 day, 3 days, 7 days, 6 weeks, 3 months, 6 months, and 12 months postoperatively. No patient was lost to follow-up or died during the follow-up period.

The primary outcome variables were the PROMs [Hip disability and Osteoarthritis Outcome Score (HOOS), patients’ hip pain on mobilization], whereas the secondary outcome variables were the Harris Hip Score (HHS), operative time, intraoperative blood loss, acetabular cup orientations (abduction, or anteversion angle), stem orientation (valgus, neutral, or varus), and the incidence of complications (intraoperative fracture, nerve damage, incisional problem, or postoperative dislocation).

One of the authors (Z.Y.) who was blinded to the group assignments evaluated all of patients. The patients’ hip pain was evaluated preoperatively and 1 day, 3 days, 7 days, 6 weeks, 3 months, 6 months, and 12 months postoperatively using a visual analog scale (VAS) from 0 (no pain) to 100 (unbearable pain). HOOS and HHS were evaluated preoperatively and 6 weeks, 3 months, 6 months, and 12 months postoperatively.

### Statistical analysis

STATA version 11.0 for Windows (StataCorp LP, College Station TX) was used for statistical analysis. The normality of continuous data was examined by the Kolmogorov–Smirnov test. The normally distributed continuous data of the two groups of patients were analyzed using paired *t*-test, and the two-sided nonparametric Wilcoxon rank-sum test was performed because the continuous data were not normally distributed. The *χ*^2^ or Fisher’s exact test was performed for the comparison of categorical data. Primary follow-up outcomes such as VSS, HOOS, and HSS were analyzed using repeated-measure analysis of variance (ANOVA). The significance level was set at *p* < 0.05.

## Results

**Fig. 1:**
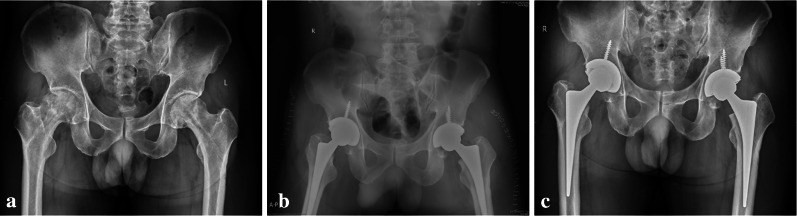
**a** Preoperative anteroposterior (AP) hip radiograph of 49-year-old male patient with bilateral femoral head osteonecrosis; **b** X‐ray images 3 days postoperatively showing hip prosthesis in a good position (right: DAA-THA; left: PLA-THA); **c** AP hip radiograph at 12-month follow-up showing the prosthesis in a good position without loosening

All 20 patients were followed up for 12 months. During the follow-up period, no patient was lost to follow-up and none died. The 20 patients (40 hips) were included in the final analysis. At final follow-up, no patients required reoperation and no radiographic loosening occurred in any hips (Fig. 1).

Demographic and baseline data for the DAA-THA and PLA-THA groups are summarized in Table 1. There was no statistically significant difference in any parameters between the groups.

**Table 1:** Detailed demographic data of patients

Parameter	DAA-THA	PLA-THA
Number of patients (hips)	20 (20)	20 (20)
Male/female	15/5	15/5
Age (years)	49.4 ± 13.3 (25–72)	49.4 ± 13.3 (25–72)
Weight (kg)	66.3 ± 9.7 (51–85)	66.3 ± 9.7 (51–85)
Height (cm)	165.6 ± 8.2 (145–178)	165.6 ± 8.2 (145–178)
Body mass index (kg/m^2^)	24.1 ± 2.6 (19.5–28.7)	24.1 ± 2.6 (19.5–28.7)
ASA class (*n*)		
I/II	13/7	13/7

The operative time for PLA-THA (53.2 ± 8.9 min) was less than that for DAA-THA (62.4 ± 9.9 min) (*p* = 0.004) (Table 2). The length of incision for DAA (10.3 ± 1.3 cm) was less than that for PLA (12.9 ± 1.6 cm) (*p* < 0.001) (Table 2). There was no statistically significant difference between PLA-THA and DAA-THA in terms of intraoperative blood loss (*p* = 0.112), abduction angle (*p* = 0.519), anteversion angle (*p* = 0.493), stem orientation (*p* = 0.133), or other complication (*p* = 1.000) (Table 2). One patient with DAA-THA had a femoral calcar fracture, which was treated by placement of cerclage wire. During the follow-up period, this patient did not experience stem loosening or subsidence.

**Table 2:** Intraoperative data, prosthesis position, and complications in the two groups

Parameter	DAA-THA	PLA-THA	*p*-Value
Operative time (min)	62.4 ± 9.9 (46–85)	53.2 ± 8.9 (42–69)	0.004
Length of incision (cm)	10.3 ± 1.3 (9–12)	12.9 ± 1.6 (11–17)	< 0.001
Intraoperative blood loss (mL)	167.4 ± 41.2 (99–254)	150.0 ± 24.4 (115–205)	0.112
Cup orientation (^o^)			
Abduction	41.7 ± 5.1 (28–47)	40.7 ± 4.6 (31–46)	0.519
Anteversion	19.0 ± 3.8 (15–28)	19.8 ± 3.5 (15–29)	0.493
Stem orientation (*n*)			
Valgus/neutral/varus	0/17/3	3/16/1	0.133
Complication (*n*)	1	0	1.000
Intraoperative fracture	1	0	
Lateral femoral cutaneous nerve damage	0		
Incision problem	0	0	
Dislocation	0	0	

Table 3 shows that, at 1, 3, and 7 days postoperatively, patients with DAA-THA reported less hip pain than those with PLA-THA (*p* < 0.05). There was no statistically significantly difference after 6 weeks, 3 ,months, 6 months, and 12 months postoperatively (*p* > 0.05).

**Table 3:** VAS of patients’ hip pain on mobilization in the two groups

Score	DAA-THA	PLA-THA	*p*-Value
Preoperatively	67.0 ± 11.7 (45–87)	65.7 ± 13.1 (44–85)	0.752
Postoperative day 1	31.6 ± 3.5 (26–38)	34.2 ± 4.5 (25–43)	0.046
Postoperative day 3	28.2 ± 2.3 (25–35)	30.6 ± 2.4 (26–35)	0.002
Postoperative day 7	21.6 ± 1.8 (19–25)	23.7 ± 2.8 (17–28)	0.008
Postoperative week 6	12.1 ± 2.4 (9–16)	12.2 ± 1.9 (8–15)	0.872
Postoperative month 3	5.3 ± 3.3 (0–10)	5.4 ± 2.2 (0–10)	0.911
Postoperative month 6	3.4 ± 2.2 (0–8)	3.5 ± 1.8 (0–7)	0.812
Postoperative month 12	0.7 ± 1.0 (0–3)	0.8 ± 1.0 (0–3)	0.872

Table 4 shows that there was no statistically significant difference in HOOS between the two groups after 6 weeks, 3 months, 6 months, and 12 months postoperatively (*p* > 0.05). Table 5 shows that there was no statistically significant difference in HSS between the two groups after 6 weeks, 3 months, 6 months, and 12 months postoperatively (*p* > 0.05).

**Table 4:** HOOS of the two groups

Score	DAA-THA	PLA-THA	*p*-Value
Preoperatively	41.6 ± 9.4 (29–64)	43.1 ± 8.9 (27–57)	0.621
Postoperative week 6	80.2 ± 11.1 (55–100)	77.0 ± 12.7 (54–97)	0.410
Postoperative month 3	85.9 ± 9.4 (65–97)	86.3 ± 8.2 (73–97)	0.901
Postoperative month 6	91.2 ± 5.4 (82–97)	92.2 ± 5.2 (77–97)	0.536
Postoperative month 12	97.6 ± 2.9 (90–100)	98.0 ± 2.7 (91–100)	0.658

**Table 5:** HSS of the two groups

Score	DAA-THA	PLA-THA	*p*-Value
Preoperatively	40.4 ± 9.5 (27–63)	41.8 ± 8.9 (26–56)	0.633
Postoperative week 6	82.9 ± 10.6 (58–100)	80.0 ± 12.7(57–100)	0.446
Postoperative month 3	88.9 ± 9.4 (68–100)	89.3 ± 8.2 (76–100)	0.901
Postoperative month 6	94.2 ± 5.4 (85–100)	95.2 ± 5.2 (80–100)	0.536
Postoperative month 12	98.7 ± 1.9 (93–100)	98.7 ± 1.9 (93–100)	1.000

## Discussion

In this study, the outcomes of DAA-THA and PLA-THA in a randomized trial of patients who underwent same-day simultaneous bilateral THA were compared, and the four most important findings were: (1) compared with PLA-THA, DAA-THA resulted in significantly less hip pain at 1, 3, and 7 days postoperatively (*p* < 0.05); (2) at 6 weeks, 3 months, 6 months, and 12 months postoperatively, there was no clinical difference between DAA-THA and PLA-THA in terms of VAS, HOOS, or HSS (*p* > 0.05); (3) compared with PLA-THA, DAA-THA had a longer operative time and shorter length of incision; (4) perioperative complications of DAA-THA and PLA-THA were similar (*p* > 0.05).

This study demonstrates that hip pain is significantly reduced with DAA-THA compared with PLA-THA at 1, 3, and 7 days postoperatively, but both are almost the same at 6 weeks, 3 months, 6 months, and 12 months postoperatively. This may be attributed to the fact that DAA minimizes muscle damage through the utilization of an interneural and intermuscular plane, which may reduce hip pain in the early postoperative period [23, 24]. Zhao et al. [23] reported lower pain on postoperative days 1–3 days with DAA compared with PLA, which causes more soft-tissue dissection and muscle damage. Mjaaland et al. [24] reported that DAA-THA resulted in less pain at 1–4 days postoperatively, which may be related to the lower damage to the muscle, or may be due to the fact that DAA does not separate the muscle attachment point. Muscle detachment from the bone is an important factor in causing pain, and the reattachment may also cause pain. After muscle reattachment and healing, postoperative pain is significantly reduced. This process is usually completed within 3–6 weeks. Thus, DAA-THA and PLA-THA have similar hip pain at 6 weeks postoperatively and thereafter.

There was no difference between DAA-THA and PLA-THA in terms of HOOS or HSS at 6 weeks, 3 months, 6 months, and 12 months postoperatively. In support of the findings, a single-institution prospective comparative study by Rodriguez et al. [25] of 120 patients with DAA-THA or PLA-THA found that, at 2-, 6-, and 12-week and 1-year postoperative follow-up, DAA-THA and PLA-THA PROMs (SF-12 scores, UCLA activity score, motor component of Functional Independence Measure) and HHS were not significantly different. Mayr et al. [26] conducted a prospective randomized study of direct anterior versus anterolateral approach to THA and showed that, at 6- and 12-week postoperative follow-up, there were no significant Western Ontario and McMaster Universities Osteoarthritis Index (WOMAC) differences between the DAA and anterolateral approach in terms of pain, stiffness, and function.

This study demonstrates a longer operative time and shorter length of incision for DAA-THA compared with PLA-THA. Recently, two literature reviews showed that DAA requires a longer operative time than PLA in THA [27, 28]. We believe that the prolonged operative time in DAA is mainly related to the difficulty of proximal femur exposure because of the need for gradual release, exposure, and the additional steps, all of which result in a longer operative time in DAA than in PLA. Although some studies have shown similar incision length for DAA and PLA [28], our results showed longer incision length for PLA than for DAA, which may be related to our surgical concept that we believe the length of skin incision does not correlate exactly with minimally invasive and does not affect clinical outcome. Therefore, we did not deliberately pursue a small skin incision of PLA.

In this study, no statistical difference was detected between the hips with a DAA-THA and PLA-THA in terms of perioperative complications. The incidence of perioperative complications in our patients with THA was low. Only one hip with DAA-THA had an intraoperative femoral calcar fracture, while no complication occurred with PLA-THA. Several studies have shown that perioperative complications are higher with DAA-THA than with PLA-THA [7], because DAA-THA has a steep learning curve. Our patients have fewer complications, either because we have surpassed the initial learning curve, or because we have a relatively small number of patients. Some studies have shown that the postoperative dislocation is higher in PLA-THA than in DAA-THA [28, 29]; however, in our study, there was no postoperative dislocation in either DAA-THA or PLA-THA. We believe that postoperative dislocation is mainly related to the position of the prosthesis, but not much to the surgical approach. Our THA was performed by an experienced surgeon, so the rate of postoperative dislocation was low.

The present study has several limitations. First, the small sample size and the results of the single surgeon studied limit the strength of the evidence. However, the selection of patients to receive same-day simultaneous bilateral THA using identical prosthetic designs reduced our patient population. Also, this selection criterion is a strength of this study. Second, the results of the study were evaluated at a 12-month follow-up, which may be too early. However, most studies have shown that the difference in efficacy between DAA-THA and PLA-THA is apparent mainly in the early postoperative period, with similar clinical outcomes at 3 months postoperatively and thereafter [3, 19]. Nevertheless, further follow-up is reasonable to determine whether the long-term clinical outcomes of DAA-THA and PLA-THA are similar. Third, the difference in the number of cases completed by the surgeon (G.C.Z.) for both surgical approaches, with a ratio of 1000:120 for PLA and DAA, may lead to bias in the analysis of outcomes. In fact, various studies have reported that DAA-THA has a learning curve of approximately 30–50 cases [30, 31], and surgeons may be proficient in operating DAA-THA after 70–100 cases [32, 33]. Therefore, the surgeon (G.C.Z.) is an expert in both approaches. Since the surgeon (G.C.Z.) has extensive surgical experience with both approaches, we believe that there may not be a huge bias in the analysis of the results.

## Conclusions

In conclusion, the results of this study show that both DAA-THA and PLA-THA provide comparable HHS and HOOS at all follow-ups. In contrast to PLA-THA, DAA-THA is associated with less hip pain within postoperative 7 days and shorter incision length, but longer operative time. The choice of the approach depends on the surgeon’s preference and experience, as DAA and PLA are equally safe and feasible for THA.

## Data Availability

The datasets used or analyzed during the current study are available from the corresponding author on reasonable request.

## Abbreviations

DAA: Direct anterior approach
PLA: Posterolateral approach
THA: Total hip arthroplasty
HOOS: Hip disability and Osteoarthritis Outcome Score
HHS: Harris Hip Score
VAS: Visual analog scale
